# Artificial Control of Giant Converse Magnetoelectric Effect in Spintronic Multiferroic Heterostructure

**DOI:** 10.1002/advs.202413566

**Published:** 2024-12-25

**Authors:** Takamasa Usami, Yuya Sanada, Shumpei Fujii, Shinya Yamada, Yu Shiratsuchi, Ryoichi Nakatani, Kohei Hamaya

**Affiliations:** ^1^ Center for Spintronics Research Network Graduate School of Engineering Science Osaka University 1‐3 Machikaneyama Toyonaka Osaka 560‐8531 Japan; ^2^ Department of Systems Innovation Graduate School of Engineering Science Osaka University 1‐3 Machikaneyama Toyonaka Osaka 560‐8531 Japan; ^3^ Spintronics Research Network Division Institute for Open and Transdisciplinary Research Initiatives Osaka University 2‐1 Yamadaoka Suita Osaka 565‐0871 Japan; ^4^ Department of Materials Science and Engineering Graduate School of Engineering Osaka University 2‐1 Yamadaoka Suita Osaka 565‐0871 Japan

**Keywords:** converse magnetoelectric effect, Heusler alloys, multiferroic heterostructures, PMN‐PT

## Abstract

To develop voltage‐controlled magnetization switching technologies for spintronics applications, a highly (422)‐oriented Co_2_FeSi layer on top of the piezoelectric PMN‐PT(011) is experimentally demonstrated by inserting a vanadium (V) ultra‐thin layer. The strength of the growth‐induced magnetic anisotropy of the (422)‐oriented Co_2_FeSi layers can be artificially controlled by tuning the thicknesses of the inserted V and the grown Co_2_FeSi layers. As a result, a giant converse magnetoelectric effect (over 10^−5^ s m^−1^) and a non‐volatile binary state at zero electric field are simultaneously achieved in the (422)‐oriented Co_2_FeSi/V/PMN‐PT(011) multiferroic heterostructure. This study leads to a way toward magnetoresistive random‐access‐memory (MRAM) with a low power writing technology.

## Graphical abstract

Voltage‐controlled magnetization switching based on the converse magnetoelectric effect is one of the key technologies for future spintronic memory devices. In this study, highly (422)‐oriented Co_2_FeSi/V/PMN‐PT multiferroic heterostructures is experimentally developed with giant converse magnetoelectric effect and controlled nonvolatile binary states.

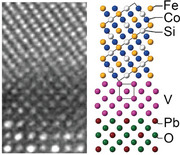

## 1 Introduction

Magnetoresistive random‐access‐memory (MRAM) is an emerging non‐volatile memory with advantageous features such as fast operation, high storage capacity, high endurance, and compatibility with CMOS technology.^[^ ^1^, ^2^, ^3^, ^4^ ^]^ In MRAM, the data is generally stored in magnetization vector configurations of magnetic tunnel junctions (MTJs), and efficient switching of these vectors is required to allow for low‐power data writing. In initial toggle MRAM devices, the data writing was performed by the Oersted field generated by the current passing through bit and digit lines.^[^ ^5^, ^6^ ^]^ However, the scheme was unsuitable for the device scaling, since the switching current increased as the memory unit cell decreased.^[^ ^4^, ^7^ ^]^ In this context, recent commercial MRAM uses the spin‐transfer torque (STT) effect injecting an electric current to switch the magnetization vectors in MTJs.^[^ ^8^, ^9^, ^10^, ^11^ ^]^ To switch the magnetization vector of MTJs at high speed, the spin‐orbit torque (SOT) scheme has also been intensively studied as a new data writing one.^[^ ^12^, ^13^, ^14^ ^]^ In this scheme, the electric current is used through the heavy metals such as Pt, W, and Ta with relatively high resistivities.^[^ ^12^, ^13^, ^15^, ^16^ ^]^

Thus far, electric‐field (*E*) control of the direction of the magnetization vectors has been studied as a means of reducing the power inputs required for data writing in MRAM.^[^ ^17^, ^18^ ^]^ As pioneering challenges, ferromagnetic semiconductors^[^ ^19^, ^20^ ^]^ and ultrathin ferromagnets on a dielectric material^[^ ^21^, ^22^ ^]^ have been explored. For practical applications, however, it is very desirable to switch the magnetization vectors by an *E* without using an assist‐magnetic field (*H*) above room temperature. Therefore, as an alternative approach, multiferroic heterostructures consisting of ferromagnetic (FM)/piezoelectric layers have been explored due to the robust operation above room temperature and a wide variety of material selections.^[^ ^23^, ^24^, ^25^, ^26^, ^27^, ^28^, ^29^, ^30^ ^]^ For example, magnetoelectric (ME)‐MRAM devices^[^ ^31^ ^]^ and a related logic device^[^ ^32^ ^]^ have been proposed, which are expected that the data writing energy will be significantly reduced. Furthermore, *E* manipulation of an antiferromagnetic order using the multiferroic heterostructures has been recently discussed.^[^ ^33^, ^34^, ^35^ ^]^

Very recently, by inserting a 0.3‐nm‐thick Fe layer at the interface, we demonstrated a giant converse magnetoelectric (CME) coupling coefficient (*α*_E_) of over 10^−5^ s m^−1^ in a multiferroic heterostructure composed of FM Heusler‐alloy Co_2_FeSi and Pb(Mg_1/3_Nb_2/3_)O_3_‐PbTiO_3_(011) [PMN‐PT(011)].^[^ ^36^ ^]^ Then, it was clarified that the giant CME effect originated from the strain‐induced modulation of the Fe sites in the (422)‐oriented Co_2_FeSi layer on the PMN‐PT(011).^[^ ^37^ ^]^ This means that obtaining highly (422)‐oriented Co_2_FeSi layers on PMN‐PT(011) is a crucial design rule to achieve the giant *α*_E_ value in the Co_2_FeSi/Fe/PMN‐PT(011) multiferroic heterostructure,^[^ ^37^ ^]^ but because of the presence of in‐plane structural fluctuations with some variants, only polycrystalline Co_2_FeSi layers have been obtained on PMN‐PT(011)^[^ ^36^ ^]^ To incorporate the multiferroic heterostructure into practical devices in future, the next two requirements should be simultaneously satisfied; the FM layer is a highly spin‐polarized magnetization free one having a giant *α*_E_ of over 10^−5^ s m^−1^ and shows a non‐volatile binary state at zero electric field. However, the presence of the in‐plane structural fluctuations has so far made it more challenging to reliably achieve almost the same magnetic anisotropy and non‐volatile binary state at the zero electric field.

In this research article, we experimentally propose the insertion of a vanadium (V) ultra‐thin layer between the Co_2_FeSi and the PMN‐PT(011) interface,^[^ ^38^ ^]^ leading to the highly (422)‐oriented Co_2_FeSi layer on top of the PMN‐PT(011). The strength of the growth induced magnetic anisotropy of the (422)‐oriented Co_2_FeSi layers can be artificially controlled by adjusting the thickness of the inserted V and the grown Co_2_FeSi layers. As a result, we simultaneously achieve a giant CME effect with a *α*_E_ of over 10^−5^ s m^−1^ and a non‐volatile binary state at zero electric field in a (422)‐oriented Co_2_FeSi/V/PMN‐PT(011) multiferroic heterostructure.

## 2 Growth and Measurements

Our previous study revealed that the growth of the high‐index (422) plane in Co_2_FeSi results in substantial modulation of Fe orbital magnetic moments, leading to the giant CME effect.^[^ ^37^ ^]^ Therefore, achieving highly oriented (422) Co_2_FeSi is crucial for obtaining giant and reliable CME effect. To promote the (422) oriented growth of Co_2_FeSi, we attempt to insert a V layer between Co_2_FeSi and PMN‐PT(011). Co_2_FeSi and V layers were grown on PMN‐PT(011) substrates by molecular beam epitaxy (MBE) techniques, where the composition of PMN‐PT is Pb(Mg_1/3_Nb_2/3_)_(1 − *x*)_O_3_‐PbTi_*x*_O_3_ (*x* = 0.29 − 0.32), close to the morphotropic phase boundary showing the large piezoelectricity.^[^ ^39^ ^]^ This is favorable for inducing the strain‐mediated CME effect and other spintronic applications.^[^ ^31^, ^32^, ^40^ ^]^ Flat and clean substrate surfaces were obtained by heat treatment (400°C for 20 min.) prior to the film growth. Note that the heat‐treatment temperature was sufficiently lower than that in the literature (∼800°C) showing a giant CME effect,^[^ ^41^ ^]^ which means that any loss of piezoelectricity would be negligible. After cooling each substrate to 300°C, a V layer with a thickness (*t*_V_) from 0.3 to 2 nm was grown. Subsequently, a Co_2_FeSi film with a thickness (*t*_CFS_) of 10, 20, and 30 nm was grown at 300°C by co‐evaporation employing Knudsen cells.^[^ ^42^, ^43^, ^44^ ^]^

Structural evaluation was performed by X‐ray diffraction (XRD) to collect the out‐of‐plane profile and the polar figures. We used an X‐ray diffractometer with Cu Kα radiation at the emission current of 200 mA and the acceleration voltage of 45 kV. In addition, scanning transmission electron microscopy (STEM) analysis with the zone axis along PMN‐PT[100] was carried out. Magnetization data were acquired under *H* along the PMN‐PT[100] and [01$\bar{1}$] using a vibrating sample magnetometer (VSM) at room temperature. The CME effect was characterized by longitudinal magneto‐optic Kerr effect (MOKE) measurements with the application of an *E*, where this method has been established in previous studies.^[^ ^41^, ^45^ ^]^ We used a conventional MOKE measurement system with an LED light source, where the wavelength was 670 nm, the beam size was about ϕ 0.7 mm, and the incident angle was about 25°.

**Figure 1:**
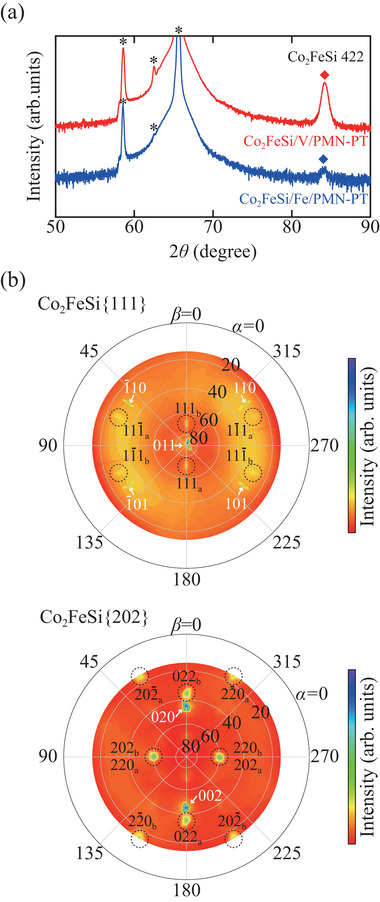
a) Out‐of‐plane XRD profiles for the 30‐nm‐thick Co_2_FeSi/PMN‐PT(011) heterostructure with inserting a 0.3‐nm‐thick V layer (red), together with that with inserting a 0.3‐nm‐thick Fe layer (blue). The denoted peaks by the diamonds and asterisks originate from the Co_2_FeSi(422) plane and PMN‐PT substrate, respectively. b) The XRD pole figures of the Co_2_FeSi/V/PMN‐PT(011) heterostructure for 111 and 202 diffractions for Co_2_FeSi. The black dashed circles indicate the diffraction spots regarding Co_2_FeSi. Black and white labels indicate the indices for Co_2_FeSi and PMN‐PT, respectively, where the subscripts show the variants contributing to the diffraction.

**Figure** **1**a shows an XRD *ω* − 2*θ* profile for a representative Co_2_FeSi(30 nm)/V(0.3 nm)/PMN‐PT(011) heterostructure. Here, the profile for the Co_2_FeSi(30 nm)/Fe(0.3 nm)/PMN‐PT(011) heterostructure in the previous work^[^ ^36^ ^]^ is also shown as a reference. For the Co_2_FeSi/V/PMN‐PT(011) heterostructure, a clear diffraction peak only from the Co_2_FeSi(422) is found at 2*θ* ≈ 84° except for the diffraction from the substrate. In addition, any secondary phases were not observed in a broader 2*θ* range (not shown here). It is worth noting that the peak intensity is significantly larger than that for the Co_2_FeSi/Fe/PMN‐PT(011) heterostructure. This means that the replacement of the insertion layer from Fe to V is quite effective to obtain (422)‐oriented Co_2_FeSi layer on PMN‐PT(011).

We further performed pole‐figure measurements for the Co_2_FeSi/V/PMN‐PT(011) heterostructure. Here, 2*θ* angles were fixed at 27.42 and 45.54 degrees for obtaining Co_2_FeSi 111 and 202 diffractions, respectively. Figure 1b shows the measured pole figures. *α* and *β* are polar and azimuthal angles, respectively, and *β* = 0 corresponds to the PMN‐PT[01$\bar{1}$] direction. As shown in the top of Figure 1b, clear Co_2_FeSi 111 diffraction spots are observed, showing the presence of the *L*2_1_‐ordered structure of the Heusler alloy. Thus, as well as the Fe layer,^[^ ^36^ ^]^ the V layer is also effective for obtaining the *L*2_1_‐ordered structure on PMN‐PT substrates. The bottom of Figure 1b shows the diffraction spots from Co_2_FeSi {202} planes. Two diffraction spots of crystallographically equivalent 022_a_ and 022_b_ are observed at *β* = 180 and 0 degree, respectively. This indicates the existence of 180‐degree‐rotated variants with two epitaxial relationships of Co_2_FeSi(422)[1$\bar{1}\bar{1}]||\mathrm{PMN}$‐PT(011)[01$\bar{1}$](variant A) and Co_2_FeSi(422)[$\bar{1}11]||\mathrm{PMN}$‐PT(011)[01$\bar{1}$] (variant B). In addition, the sharp diffraction spots of 202_a_ and 220_b_ (220_a_ and 202_b_) are seen in the same position, showing the absence of the in‐plane structural fluctuations of the grown Co_2_FeSi. In contrast, the diffraction spots in the Co_2_FeSi/Fe/PMN‐PT(011) heterostructure are divided into two spots.^[^ ^36^ ^]^ From these results, the insertion of the V layer plays an important role in obtaining the (422)‐oriented Co_2_FeSi without forming the in‐plane structural fluctuations.

**Figure 2:**
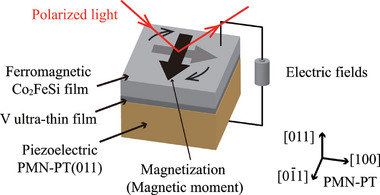
Schematic of the Co_2_FeSi/V/PMN‐PT(011) heterostructures. Electric fields are applied perpendicular to the plane of the Co_2_FeSi/V/PMN‐PT heterostructure.

To evaluate the CME effect for the Co_2_FeSi/V/PMN‐PT heterostructures, an Au(100 nm)/Ti (3 nm) backside electrode was deposited on the PMN‐PT, while a Co_2_FeSi film was used as the top electrode. The prepared structure enables us to apply an *E* perpendicular to the plane of the Co_2_FeSi/V/PMN‐PT heterostructure. Under the application of the *E*, the *H* dependence of the Kerr ellipticity was measured at room temperature, where an in‐plane external *H* was applied. A schematic of the Co_2_FeSi/V/PMN‐PT sample and the experimental setup for evaluating the CME effect is illustrated in **Figure** **2**.

## 3 Results and Discussion

### 3.1 Highly (422)‐Oriented Co_2_FeSi

**Figure 3:**
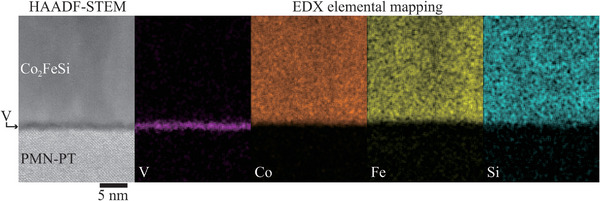
High‐angle annular dark‐field scanning transmission electron microscope (HAADF‐STEM) image and energy dispersive X‐ray spectroscopy (EDX) elemental maps for a Co_2_FeSi/V/PMN‐PT(011) heterostructure, where the zone axis is PMN‐PT[100].

In **Figure** **3**, we show a cross‐sectional high‐angle annular dark‐field scanning transmission electron microscope (HAADF‐STEM) image and energy dispersive X‐ray spectroscopy (EDX) maps for a Co_2_FeSi/V/PMN‐PT(011) heterostructure, where *t*_V_ estimated from the deposition time is 0.6 nm. We find that, from the EDX maps, a homogeneous and single‐phase Co_2_FeSi layer is formed over the measured area. The chemical composition of the Co_2_FeSi layer is confirmed to be almost stoichiometric (Co: Fe: Si = 2.09: 0.96: 0.95). Notably, the V layer stays at the interface between the Co_2_FeSi and PMN‐PT substrate. Because the atomic number of V is relatively small compared to Co, Fe, and Pb, the HAADF‐STEM image of the interface between the Co_2_FeSi layer and PMN‐PT substrate becomes dark, being reasonable as an expected Co_2_FeSi/V/PMN‐PT(011) heterostructure. Furthermore, an element‐dependent EDX line profile across the interface also revealed that the V layer remained only at the interface within 2 nm (not shown here).

**Figure 4:**
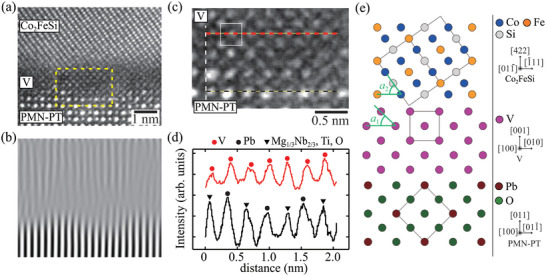
a) Enlarged HAADF‐STEM image for the interface of the Co_2_FeSi/V/PMN‐PT(011) heterostructure with the zone axis, PMN‐PT[100]. b) The corresponding Fourier filtered diffraction image. c) An enlarged HAADF‐STEM image regarding the V/PMN‐PT interface. The area of the image corresponds to the dashed yellow box in (a), and the zone axis is along PMN‐PT[100]. d) The intensity line profiles along the red and black dashed lines in (c). e) Schematic of possible atomic arrangements at the Co_2_FeSi/V/PMN‐PT(011) heterostructure. Here, the Mg, Nb, and Ti atoms are not displayed in the schematic of PMN‐PT, because they are located behind the O atoms.

To get insight to the V‐inserted interface between Co_2_FeSi and PMN‐PT substrate, we further characterize high‐resolution analyses for an enlarged HAADF‐STEM image in **Figure** **4**a. Although an unclear interface partially exists, a layered structure of Co_2_FeSi and V lattices on PMN‐PT can be observed. This is largely different from the result that an oxidized layer existed at the interface of the Co_2_FeSi/Fe/PMN‐PT(011) heterostructure in the previous work.^[^ ^36^ ^]^ Namely, the insertion of the ultra‐thin V layer between Co_2_FeSi and PMN‐PT is significantly effective to improve the interface quality. Figure 4b shows the Fourier‐filtered diffraction image corresponding to the HAADF‐STEM one (Figure 4a). Although there are some dislocations, the continuous lines partly exist across the Co_2_FeSi/V/PMN‐PT(011) interface. It is expected that the V layer is working as a buffer layer for the growth of Co_2_FeSi on top of the PMN‐PT(011).

To clarify this, we focus on an enlarged HAADF‐STEM image of the V layer near the interface (Figure 4c). The body‐centered cubic (bcc) structure is clearly observed, as indicated by the white squares. This means that an ultra‐thin (001)‐oriented V layer is basically grown on the PMN‐PT(011). For further investigation of the structure, intensity profiles regarding the V layer (red) and the PMN‐PT substrate (black) are investigated from the red and black dashed lines in Figure 4c. The intensity profiles are shown in Figure 4d. As a result, the position of the V atoms on the red dashed line nearly corresponds to the atoms of the surface of the PMN‐PT substrate on the black dashed line. Considering these results, we infer that the (001)‐oriented V layer is epitaxially grown on the PMN‐PT(011), as schematically shown in Figure 4e. In the schematic, the angles of *a*_1_ and *a*_2_ are 45.0 and 54.7 degrees, respectively. A similar difference in the angles can also be observed in the HAADF‐STEM image in Figure 4a. From these features of the V layer, we experimentally obtain highly (422)‐oriented Co_2_FeSi even on PMN‐PT(011) substrates.

### 3.2 Control of Giant CME Effect

Using the highly (422)‐oriented Co_2_FeSi/V/PMN‐PT(011) multiferroic heterostructures, we focus on the establishment of a reliable and non‐volatile binary magnetic state. According to the previous study by Wang et al.,^[^ ^46^ ^]^ the non‐volatile binary magnetic states were controlled in ferromagnet/PMN‐PT(011) multiferroic heterostructures. In general, it is important to consider the competition between the growth‐induced magnetic anisotropy energy (*E*_g_ = *K*_g_sin ^2^ *θ*) and the magnetoelastic energy (*E*_u_ = *K*_u_cos ^2^ *θ*), where *K*_g_ is the growth‐induced anisotropy constant, *K*_u_ is the magnetoelastic energy constant arising from the anisotropic strain ($\varepsilon_{\left[100\right]}-\varepsilon_{\left[01\bar{1}\right]}$) of PMN‐PT. *θ* is defined as the relative angle between the in‐plane magnetization vector and the PMN‐PT[01$\bar{1}$] direction in this study. Note that *K*_g_ > 0 means the growth‐induced magnetic anisotropy provides the magnetic easy axis (MEA) along PMN‐PT[01$\bar{1}$]. On the other hand, *K*_u_ > 0 means the magnetoelastic energy induces the MEA along PMN‐PT[100]. As Wang et al. pointed out, it is important to consider the strength of the *K*_g_ value to tune the resulting total energy density, (*K*_u_ − *K*_g_)cos ^2^ *θ*.^[^ ^46^ ^]^ In other word, for *K*_u_ > *K*_g_, the MEA was aligned along to PMN‐PT[100], while for *K*_u_ < *K*_g_, the MEA was controlled along to PMN‐PT[01$\bar{1}$].

On the other hand, the value of *K*_u_ can be considered to be proportional to the anisotropic strain modulated by the application of an *E* in the case of multiferroic heterostructures composed of PMN‐PT(011).^[^ ^36^, ^46^ ^]^ Since the anisotropic strain enables two states at *E* = 0, the value of *K*_u_ can also take two states. As a consequence, if *K*_g_ was a moderate strength, the total energy density, (*K*_u_ − *K*_g_)cos ^2^ *θ*, can take two states, leading to the binary magnetic states for non‐volatile memory. On the basis of the above concept, Wang *et al.* have demonstrated both the giant CME effect and the non‐volatile magnetization switching in the amorphous Co_40_Fe_40_B_20_/PMN‐PT(011) heterostructures. Unfortunately, the control of the *K*_g_ value was conducted by the application of external magnetic fields during the deposition of amorphous CoFeB films on PMN‐PT(011). Since we have shown the highly (422)‐oriented Co_2_FeSi layers on PMN‐PT(011) only by inserting ultra‐thin V layer, we should further explore a possibility to tune *K*_g_ by varying the structural configuration of Co_2_FeSi/V/PMN‐PT(011) heterostructure.^[^ ^47^ ^]^

**Figure 5:**
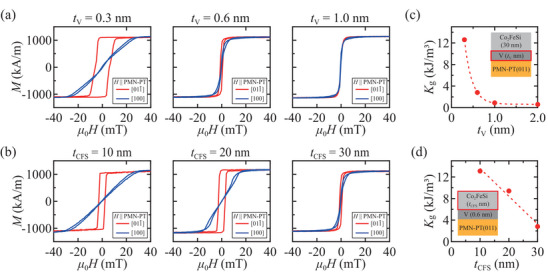
Panels (a) and (b) are representative *M*‐*H* curves at room temperature for various *t*_V_ and *t*_CFS_ in the case of Co_2_FeSi(30 nm)/V(*t*_V_ nm)/PMN‐PT(011) and Co_2_FeSi(*t*_CFS_ nm)/V(0.6 nm)/PMN‐PT(011), respectively. Panels (c) and (d) are the estimated *K*_g_ versus *t*_V_ and *t*_CFS_, respectively.

We firstly show magnetic‐field dependent magnetization (*M* − *H* curves) for Co_2_FeSi(*t*_CFS_ nm)/V(*t*_V_ nm)/PMN‐PT(011) heterostructures. In **Figure** **5**a,b, the representative *M* − *H* curves at room temperature for various *t*_V_ and *t*_CFS_ in the case of Co_2_FeSi(30 nm)/V(*t*_V_ nm)/PMN‐PT(011) and Co_2_FeSi(*t*_CFS_ nm)/V(0.6 nm)/PMN‐PT(011), respectively. We can see significant change in the growth‐induced magnetic anisotropy by changing *t*_V_ and *t*_CFS_. Notably, the value of *K*_g_ seems to be enhanced by decreasing *t*_V_ and *t*_CFS_. To quantitatively estimate the value of *K*_g_ from the *M* − *H* curves, we use the following relationship,

(1) $$K_{g}=\int_{0}^{M_{s}}\left(H_{100}-H_{01\bar{1}}\right)dM$$

where *M*_s_, $H_{01\bar{1}}$, and *H*_100_ are the saturation magnetization, the applied magnetic field along PMN‐PT[$01\bar{1}$], and that along PMN‐PT[100], respectively. Figure 5c,d presents the estimated values of *K*_g_ as a function of *t*_V_ and *t*_CFS_, respectively. The value of *K*_g_ systematically decreases with increasing *t*_V_ and *t*_CFS_. Whereas *t*_V_ dependence of *K*_g_ shows an exponential decay, *t*_CFS_ dependence of *K*_g_ shows monotonic decrease. Therefore, the effect of *t*_V_ on *K*_g_ is stronger than that of *t*_CFS_. From these results, the value of *K*_g_ is able to be controlled intentionally without applying external magnetic fields during the growth. Note that, even for the weakly (422)‐oriented Co_2_FeSi layers in the previous study, in‐plane magnetic anisotropy and its variation by the in‐plane strain are governed by the orbital magnetic moments of the Fe sites.^[^ ^37^ ^]^ In the present, study, the intensity of the 422 diffractions is significantly enhanced by inserting only a few‐nm‐thick V layer. Namely, we conclude that the magnitude of *K*_g_ and its modulation originate from the increase in the (422)‐oriented Co_2_FeSi layer by variating V and Co_2_FeSi thickness.

**Figure 6:**
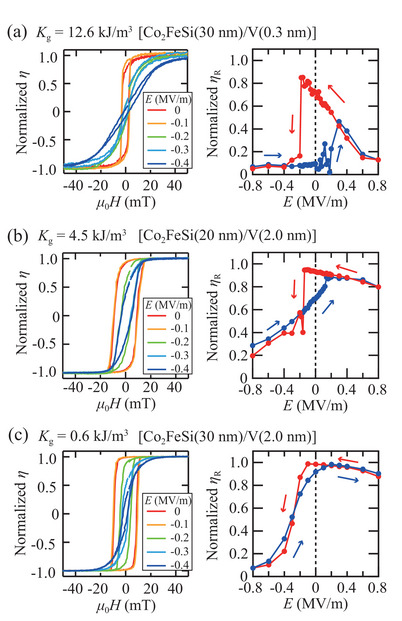
The normalized MOKE ellipticity (*η*) at different *E* and the *E* dependence of the normalized *η* at the remanent state (*η*_R_) for the Co_2_FeSi(30 nm)/V(*t*_V_ nm)/PMN‐PT(011) heterostructures with *K*_g_ = (a)12.6, b) 4.5, and c) 0.6 kJ m^−3^. The blue and red plots present the up‐sweep and down‐sweep data, respectively, within an *E* of ±0.8 MV m^−1^.

Next, the *E*‐dependent in‐plane magneto‐optic Kerr effect is measured for all the Co_2_FeSi/V/PMN‐PT(011) heterostructures to examine the CME effect. **Figure** **6**a shows the normalized MOKE ellipticity (*η*) at various *E* (left) and the values of normalized *η* at the remanent state (*η*_R_) as a function of *E* (right) for the Co_2_FeSi(30 nm)/V(0.3 nm)/PMN‐PT(011) heterostructure (*K*_g_ = 12.6 kJ m^−3^). Here, *H* was applied along PMN‐PT[100]. The Kerr hysteresis loops markedly change by applying *E*, meaning the evident CME effect at room temperature. It should be noted that, in the right panel, we can evidently observe two *η*_R_ values at *E* = 0, indicating that a distinct non‐volatile binary state is emerged. In addition, a steep change in *η*_R_ is observed at around *E* of –0.2 MV m^−1^. These features indicate that a giant CME effect and a non‐volatile binary state at zero electric field are simultaneously demonstrated in the (422)‐oriented Co_2_FeSi/V/PMN‐PT(011) multiferroic heterostructure. Notably, we confirmed that multiple samples composed of Co_2_FeSi (30 nm)/V(0.3 nm)/PMN‐PT(011)) exhibit a non‐volatile feature at *E* = 0, verifying the reproducibility and reliability of the non‐volatile binary state.

In Figure 6b,c, we also observe modulation in the Kerr hysteresis loops for the Co_2_FeSi(20 nm)/V(2.0 nm)/PMN‐PT(011) (*K*_g_ = 4.5 kJ/m^3^) and Co_2_FeSi(30 nm)/V(2.0 nm)/PMN‐PT(011) (*K*_g_ = 0.6 kJ m^−3^), respectively, by applying *E*. Here, the blue and red plots present the up‐sweep and down‐sweep data, respectively, within an *E* of ±0.8 MV m^−1^. However, the features of the normalized *η* − *E* curves are markedly varied with decreasing *K*_g_, that is, the non‐volatile binary state at *E* = 0 becomes small with decreasing *K*_g_. Namely, the *K*_g_ value of 12.6 kJ m^−3^ is a moderate one to control the value of the total energy density, (*K*_u_ − *K*_g_)cos ^2^ *θ*, as discussed in previous paragraph. In addition to the artificially controlled non‐volatile binary state, it is worth noting that steep changes of *η*_R_ at around *E* of ‐0.2 MV m^−1^ should be taken attention for the heterostructures with *K*_g_ = 12.6 and 4.5 kJ m^−3^ in Figure 6a,b, respectively. The steep changes reflect the magnetization vector switching from the PMN‐PT[100] to a certain direction with a giant *α*_E_.

The CME effect associated with the Co_2_FeSi/V/PMN‐PT(011) heterostructures is evaluated quantitatively by estimating *α*_E_ ($=|\mu_{0}\frac{dM_{R}}{\mathrm{dE}}|$), where µ_0_ is the permeability of vacuum. Here, *M*_R_ values were determind as *M*_R_ = *M*_S_(*η*_R_/*η*_S_), where *η*_S_ is *η* in the saturation state. As a result, we obtain a giant *α*_E_ of ∼2.4 × 10^−5^ s m^−1^ for the Co_2_FeSi/V/PMN‐PT(011) heterostructure with *K*_g_ = 12.6 kJ m^−3^. Notably, the observed *α*_E_ value is larger than that of the Co_2_FeSi/Fe/PMN‐PT(011) heterostructure (1.0∼1.8 × 10^−5^) in our previous works,^[^ ^36^, ^37^ ^]^ and is comparable to that reported for the magnetostrictive materials, Fe_80_Ga_20_/MgO/PMN‐PT(001) (1.5∼4.5 × 10^−5^ s m^−1^)^[^ ^41^ ^]^ and Fe_70_Ga_30_/PMN‐PT(001) (1.5∼2.0 × 10^−5^ s m^−1^).^[^ ^48^ ^]^ Therefore, the Co_2_FeSi/V/PMN‐PT(011) heterostructures simultaneously satisfies the reliable non‐volatile binary state at *E* = 0 and the giant CME effect, being promising interfacial multiferroic heterostructures for the spintronics applications including the memory devices. In future, demonstrating a multiferroic heterostructure with a thin piezoelectric layer will be essential. In such cases, precise control of *K*_g_ becomes highly important, as the magnitude of piezostrain can sensitively vary in thin piezoelectric layers, compared to bulk piezoelectric substrates. Therefore, this study provides valuable design guidelines for future progress for electric‐field‐controlled MRAM devices.

## 4 Conclusion

To establish the reliable non‐volatile binary state at *E* = 0 and the giant CME effect in interfacial multiferroic heterostructures for spintronic applications, we have focused on the control of the strength of the growth‐induced magnetic anisotropy in the highly spin polarized Co_2_FeSi on the ferroelectric PMN‐PT(011). By inserting the V layer between Co_2_FeSi and PMN‐PT(011), we have obtained highly (422)‐oriented Co_2_FeSi layers on PMN‐PT(011). Also, we have artificially controlled the strength of the growth‐induced magnetic anisotropy by tuning the thicknesses of the inserted V and the grown Co_2_FeSi layers. As a result, we found a design guideline to achieve the giant CME effect (over 10^−5^ s m^−1^) and non‐volatile binary state at zero electric field in the (422)‐oriented Co_2_FeSi/V/PMN‐PT(011) multiferroic heterostructure. This study leads to a way toward MRAM with a low power writing technology.

## Conflict of Interest

The authors declare no conflict of interest.

## Data Availability

The data that support the findings of this study are available from the corresponding author upon reasonable request.
